# The effects of MDR/RR-TB treatment on HIV disease: A systematic review of literature

**DOI:** 10.1371/journal.pone.0248174

**Published:** 2021-03-05

**Authors:** Keri Geiger, Paul D. Stamper, Jason E. Farley

**Affiliations:** School of Nursing, Johns Hopkins University, Baltimore, MD, United States of America; US Department of State Bureau of Diplomatic Security, UNITED STATES

## Abstract

**Background:**

Multidrug-resistant or rifampicin-resistant tuberculosis (MDR/RR-TB) and human immunodeficiency virus (HIV) co-infection are a deadly combination. While evidence on the effects of HIV co-infection on MDR/RR-TB treatment outcomes is well-documented, little published evidence describes the effects of MDR/RR-TB treatment on HIV disease.

**Methods:**

We conducted a review of literature published prior to June 2020. We searched Pubmed, CINAHL, and EMBASE using variations of the terms “multidrug-resistant tuberculosis,” “HIV,” and either “CD4” or “viral load.” Two reviewers independently completed title and abstract screening, full-text screening, article evaluation, and data extraction. We also included five published articles evaluated as evidence by the World Health Organization (WHO) in preparation for the 2019 MDR/RR-TB treatment guideline update.

**Results:**

A total of 459 references were returned, with 362 remaining after duplicate removal. Following article screening, six manuscripts were included. Articles reported CD4 count and/or viral load results for MDR/RR-TB and HIV co-infected patients during and/or after MDR/RR-TB treatment. The additional five references identified from the WHO guideline revision did not report HIV disease indicators after MDR/RR-TB initiation.

**Conclusion:**

There is a paucity of evidence on HIV disease indicators following MDR/RR-TB treatment. Researchers should report longitudinal HIV disease indicators in co-infected patients in publications.

## Introduction

Tuberculosis (TB) is the world’s deadliest infectious disease and the leading cause of death for people living with human immunodeficiency virus (HIV) [1]. The World Health Organization (WHO) has called multidrug-resistant or rifampicin-resistant tuberculosis (MDR/RR-TB) a “public health crisis and health security threat” [1]. The WHO estimates that there were 484,000 new cases of MDR/RR-TB in 2018 [1]. Although MDR/RR-TB is a curable disease, only about one-third of those who have MDR/RR-TB start on treatment, and of those only 56% are treated successfully [1].

HIV and MDR/RR-TB are a deadly combination that is common in some areas such as sub-Saharan Africa. Due to potential for weakened immunity, people living with HIV and AIDS (PLWHA) are more likely to progress from latent infection to active disease following exposure to TB or MDR/RR-TB [1]. The need to coordinate MDR/RR-TB and HIV treatment regimens poses clinical challenges due to drug-drug interactions [2], excessive pill burden, regimen complexity, and amplified and overlapping side effects [3]. WHO MDR/RR-TB treatment guidelines encourage switching antiretroviral treatment (ART) regimens rather than modifying MDR/RR-TB medication choice or dosage, yet these guidelines do not specify which ART regimen should be used during MDR/RR-TB treatment, and individual country-level guidelines often leave this choice to a treating clinician [4]. This situation is one of several reasons the WHO recommends integration of MDR/RR-TB and HIV services to streamline care delivery and improve outcomes for co-infected patients [5]. High-quality HIV care during MDR/RR-TB treatment is imperative to ensure patients’ ability to achieve viral suppression is not compromised.

Despite the challenges of concurrent treatment for MDR/RR-TB and HIV co-infection, MDR/RR-TB treatment includes frequent interactions between clinicians and patients, providing an opportunity for clinicians to address a patient’s engagement and retention in HIV care and for the patient to achieve viral suppression. In 2019 the WHO released new guidelines for MDR/RR-TB treatment that have changed the standard of care and have potential to improve MDR/RR-TB outcomes through an all-oral MDR/RR-TB treatment regimen with excellent efficacy against drug-resistant forms of TB [4]. However, it is not well understood how new guidelines and novel antitubercular agents affect HIV disease and its treatment.

Several systematic reviews have evaluated the incidence of MDR/RR-TB and HIV co-infection, MDR/RR-TB treatment outcomes for HIV co-infected patients, and the safety and efficacy of various treatment regimens for MDR/RR-TB in HIV-positive and HIV-negative patients [6–9]. However, these reviews focus on MDR/RR-TB indicators with limited or no discussion of how MDR/RR-TB treatment affects HIV disease. Despite the extra challenges that HIV co-infection adds to MDR/RR-TB treatment, some studies have shown similar MDR/RR-TB treatment success rates among HIV-positive and HIV-negative MDR/RR-TB patients when ART is available [10, 11]. As HIV disease management requires lifelong ART extending well beyond MDR/RR-TB cure, examining how MDR/RR-TB treatment affects HIV disease, its treatment, and its progression is crucial [3]. This systematic review of literature synthesizes the available evidence related to the effects of MDR/RR-TB treatment on HIV disease in co-infected patients by examining reported HIV indicators, CD4 count and HIV viral load, in published literature about MDR/RR-TB cohorts. Data related to HIV co-infection is also analyzed in studies related to the efficacy of newer, all-oral MDR/RR-TB treatment regimens.

## Materials and methods

We conducted a systematic review of literature designed to provide evidence about the effects of MDR/RR-TB treatment on HIV disease indicators in MDR/RR-TB and HIV co-infected patients. The databases Pubmed, EMBASE, and CINAHL were searched in September 2019 with a repeated search in May 2020 using a combination of Medical Subject Heading (MeSH) terms or their equivalent and free-text words. An information scientist specializing in medical and public health information was consulted in the development of the search terms. Search terms included variations of “multidrug-resistant tuberculosis,” “drug-resistant tuberculosis”, “rifampicin-resistant tuberculosis”, “HIV,” “viral load,” and “CD4 count.” There is no indexed MeSH term for RR-TB in any of the databases. The search was not limited by publication year or type of reference. There was no published protocol for this systematic review. The search strategy is further detailed in S1 Table.

**Fig 1:**
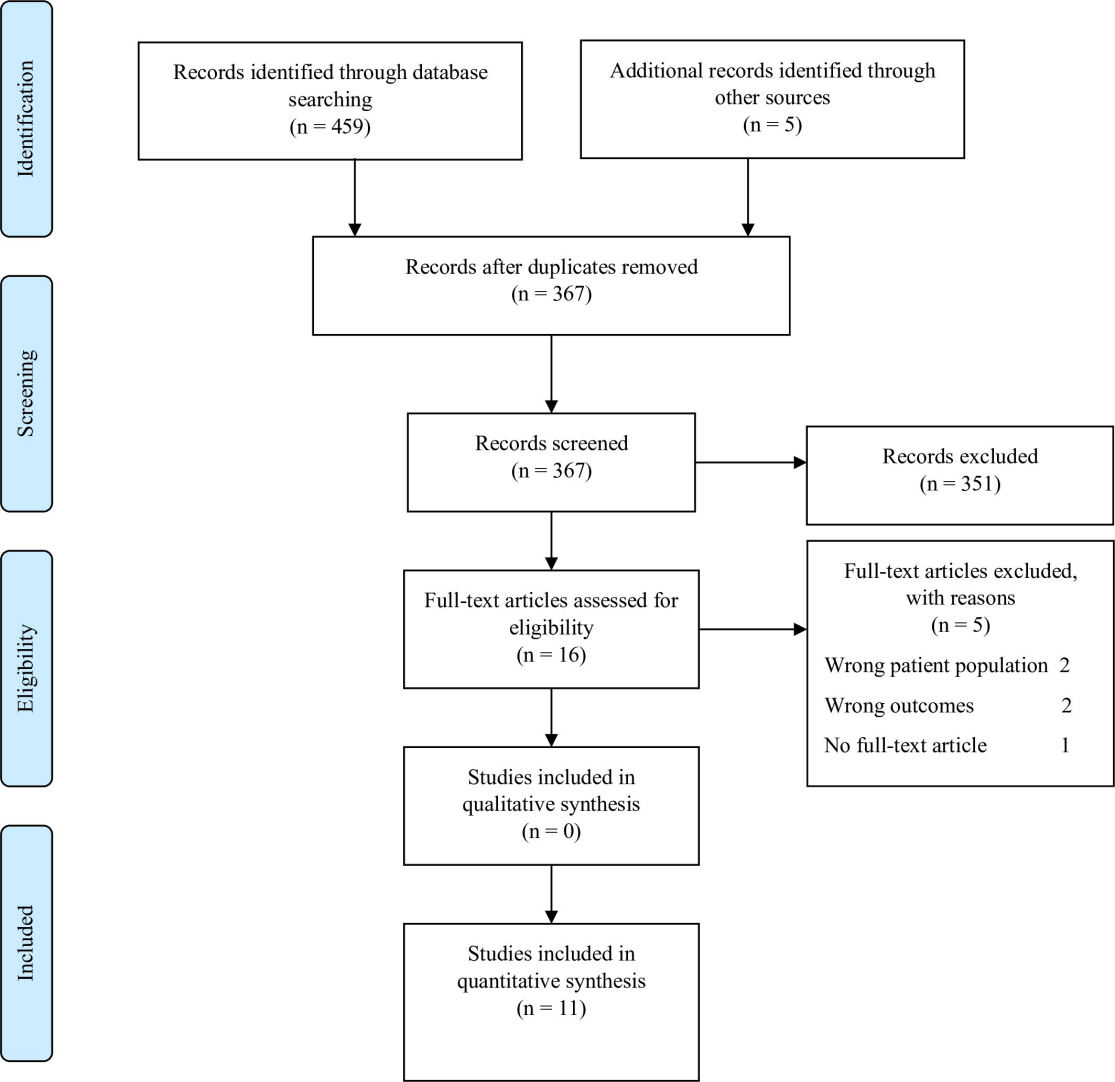
PRISMA diagram. Flow diagram of included studies.

The initial search returned a total of 452 references which were uploaded into Covidence®, a web-based software for organizing and managing references during the literature review process [12]. Inclusion criteria were intentionally broad and included: 1) inclusion of MDR/RR-TB and HIV co-infected patients and 2) discussion of HIV disease indicators. Exclusion criteria included: 1) no measurement of HIV disease indicators after MDR/RR-TB treatment initiation, 2) full-text article unavailable, 3) article not published in English, or 4) HIV disease indicators were not reported separately for MDR/RR-TB patients versus those with drug-sensitive TB. Of the original 459 references returned, 97 were duplicates. The remaining 362 references underwent title and abstract screening to determine relevance to the topic. Eleven articles progressed to full-text review, of which five were excluded due to combined reporting of HIV indicators for both drug-sensitive TB and MDR/RR-TB patients (wrong patient population, n = 2), no measurement of HIV disease-specific indicators after baseline (wrong outcomes, n = 2), or no full-text article available (n = 1). Two authors (KG and PDS) independently completed title and abstract screening as well as full-text screening and discussed discrepancies upon completion of the process. No discrepancies remained after discussion. The six remaining articles were assessed for quality using the Johanna Briggs Institute critical appraisal tools [13–19]. Fig 1 details the search results according to the Preferred Reporting Items for Systematic Reviews and Meta-analyses (PRISMA) [20].

Given the general lack of evidence and the age of the articles found in the initial search, we broadened the scope of the review to include evidence related to HIV disease in newer MDR/RR-TB cohorts. To prepare the updated MDR/RR-TB guidelines released in 2019, WHO convened a working group to review evidence on the safety and efficacy of newer and shorter treatment regimens, including regimens with the novel antimicrobial bedaquiline. We analyzed the published manuscripts describing the five cohorts included in the WHO analysis to determine what HIV-specific data was reported by the authors and how the included HIV data was reflected in the outcomes of the study [22–26].

## Results

### HIV disease indicators in injectable MDR/RR-TB treatment

Six articles, four cohort studies [14–17] and two case series [13, 18], met the inclusion criteria. These studies include MDR/RR-TB and HIV co-infected patients treated between 2008 and 2013, and all patients were treated with standard, not short course, injectable MDR/RR-TB regimens. Only four of the included articles, including the two case series, reported the ART regimens used to treat MDR/RR-TB and HIV co-infected patients during the study period [13–15, 18]. These articles noted that ART regimens were aligned with national guidelines and first-line regimens included the. medications tenofovir, stavudine, lamivudine, zidovudine with efavirenz [13–15, 18] Given the small number of patients and the types of cases described in the case series manuscripts, these two articles contribute little to answer the questions posed in this review and are discussed separately. The remaining four articles reported two HIV disease-specific indicators: CD4 cell count [14, 15, 17] and viral load [14–16].

### Evidence related to CD4 outcomes

Three of the four cohort study manuscripts, summarized in Table 1, reported CD4 measurements taken after MDR/RR-TB treatment initiation for patients on ART [14, 15, 17]. However, as CD4 improvement is time dependent and CD4 count was measured at different time points in each article, median change in CD4 count varied by study. One study reported obtaining frequent repeat CD4 counts during MDR/RR-TB treatment and up to one year following MDR/RR-TB outcome, but the results of many of these repeated measures were not reported [15]. This study did not report the proportion of HIV co-infected patients for whom a CD4 count result was available at the specified time point [15]. The remaining two articles reported CD4 measurements at MDR/RR-TB treatment initiation and one or two follow-up measures as described in Table 1, although results were not reported for all HIV co-infected participants [14, 17].

**Table 1:** CD4 count outcome.

Lead author	Year Published	Location of Study	Time period of MDR/RR-TB treatment initiation	Study design	Number of MDR/RR-TB AND HIV co-infected patients included in the study	Timing of CD4 measures (months after MDR/RR-TB treatment initiation)	Number of participants with available CD4 count at each measured time point	Median CD4 count (cells/μL)
**Brust**	2012	KwaZulu- Natal, South Africa	2/2008-3/2010	Prospective observational cohort with no control group	66	0 (baseline), 6, 12	Baseline = 48	Baseline = 170
							Month 6 = 47	Month 6 = 222
							Month 12 = 21	Month 12 = 260
**Oladimeji**	2014	Nigeria	7/2010-10/2012	Retrospective observational cohort with no control group	28	0 (baseline), 6	Baseline = 28	Baseline = 415
							Month 6 = 21	Month 6 = 491
**Brust**	2018	KwaZulu- Natal, South Africa	5/2011-12/2013	Prospective observational cohort with control group	150	0 (baseline), every 3 months following initiation until 1 year post-treatment completion (but only reported at 12 and 24 months)	Not reported at any measured time point	Baseline = 215
								Month 12 = 321
								Month 24 = 386

### Evidence related to viral load outcomes

Although HIV viral load monitoring is the primary measure of HIV treatment effectiveness, WHO guidelines have only recommended routine viral load monitoring for all patients on ART since 2013 [31]. Though all four included cohort study manuscripts reported data from cohorts that finished MDR/RR-TB treatment in 2013 or before, three manuscripts reported viral load outcomes after MDR/RR-TB treatment initiation. Each of these varied in its definition of an undetectable viral load [14–16]. As WHO defines viral suppression as a viral load below the detectable limit of the available assay [32], these variations likely resulted from different studies using different viral load assays, though study authors did not report their testing platform. These studies and their contributed evidence are summarized in Table 2.

**Table 2:** Viral load outcomes.

Lead author	Year Published	Location of Study	Time period of MDR/RR-TB treatment initiation	Study design	Number of MDR/RR-TB AND HIV co-infected patients included in the study	Timing of VL measures (months after MDR/RR-TB treatment initiation)	Number of participants with available VL count at each measured time point	Proportion of patients with documented undetectable VL
**Brust**	2012	KwaZulu Natal, South Africa	2/2008-3/2010	Prospective observational cohort with no control group	66	12	34	82%
**Efsen**	2017	Multiple countries in Eastern Europe	1/2011-12/2013	Prospective observational cohort with no control group	105	0 (baseline), 3, 7, 13, 21 (but proportion virally suppressed not reported except in aggregate)	Not reported	<20% at any time point
**Brust**	2018	KwaZulu Natal, South Africa	5/2011-12/2013	Prospective observational cohort with control group	150	0 (baseline), every 3 months following initiation until 1-year post-treatment completion (but only reported at 12 and 24 months)	Not reported at any measured time point	Baseline = 61%
								Month 12 = 76%
								Month 24 = 64%

### Evidence contributed by case series articles

Two articles that met inclusion criteria were case series [13, 18]. Both focused on MDR/RR-TB and HIV co-infected patients who were diagnosed with virologic failure and changed from first- to second-line ART during the period of MDR/RR-TB treatment. The articles reported CD4 and viral load results when the ART regimen was changed; that is, the results that led to the diagnosis of virologic failure of ART treatment. The duration of MDR/RR-TB treatment prior to the diagnosis of virologic failure differed for every individual. Thus, the results reported in these two studies were not applicable to the questions posed in this review.

### HIV disease indicators in oral MDR/RR-TB treatment

Prior to the release of updated MDR/RR-TB treatment guidelines, in both 2016 and 2018 the WHO convened a Guideline Development Group to evaluate recent evidence on the length and composition of MDR/RR-TB treatment regimens [21]. One of the systematic reviews conducted by a Guideline Development Group member in 2016 was analyzed to identify five cohorts of MDR/RR-TB patients treated with bedaquiline [22–26]. All five of these cohorts included PLWHA, though some cohorts had small numbers as shown in Table 3. As the focus of these studies was to evaluate the effectiveness of bedaquiline as part of the MDR/RR-TB regimen, none reported HIV viral load or CD4 count measurement after treatment initiation [22–26]. Rather, in two articles, HIV disease status and disease indicators were used to model MDR/RR-TB outcome [22, 24], while in the other articles, there was no further discussion of HIV disease beyond descriptive statistics at baseline.

**Table 3:** Outcomes from bedaquiline cohorts.

Lead author	Year Published	Location of Study	Beginning of recruitment	Study design	Number of MDR/RR-TB patients included in the study	Number of MDR/RR-TB AND HIV co-infected patients included in the study	HIV-specific measures included at baseline	Study findings related to HIV
**Borisov**	2017	25 centers in 15 countries	1/2008-8/2016	Retrospective observational cohort	428	94	HIV status, ART status, CD4 count	None; baseline proportion HIV positive compared across region but no HIV-specific findings addressed
**Guglielmetti**	2017	France	1/2011-12/2013	Retrospective observational cohort	102	21	HIV status	None; baseline proportion HIV positive reported for informational purposes only
**Hewison**	2018	Armenia and Georgia	4/2013-4/2015	Retrospective observational cohort	82	4	HIV status	HIV status was not a significant risk factor for unfavorable MDR/RR-TB outcome
**Ndjeka**	2018	South Africa	2012-3/2015	Retrospective observational cohort	200	134	HIV status, ART status, CD4 count, viral load	HIV status and viral load >1000 copies/mL were not significant predictors of MDR/RR-TB outcome
**Pym**	2016	11 countries	8/2009-9/2010	Prospective cohort	233	9	HIV status	None; baseline proportion HIV positive reported for informational purposes only

Only one article reported which ART regimens were used by PLWH during the study [22]. This study reported that standardized national ART regimens were used, including tenofovir, emtricitabine, and efavirenz, and patients with a drug-drug interaction between efavirenz and the oral MDR-TB medication bedaquiline were changed to either nevirapine or lopinavir-ritonavir, with no mention of the proportion of patients who had an ART substitution or the frequency with which each alternate regimen was chosen [22].

## Discussion

Although the proportion of MDR/RR-TB patients achieving cure has historically been low, the new WHO guidelines that allow for all-oral MDR/RR-TB treatment regimens and the quick response of some countries such as South Africa to adopt and implement these guidelines presents a historic moment in the MDR/RR-TB epidemic. With newer treatment regimens, more patients, including HIV co-infected patients, are expected to survive MDR/RR-TB disease. Given these great advances in MDR/RR-TB treatment and the continued burden of HIV co-infection in many parts of the world, particularly sub-Saharan Africa, shifting focus away from only MDR/RR-TB disease to include important comorbidities is appropriate. For all types of TB, evidence has shown that survivors suffer increased morbidity and mortality compared to those who have not endured a TB episode [27]. Research on post-treatment morbidity and mortality outcomes for MDR/RR-TB survivors is limited, but several studies have reported residual pulmonary sequelae after MDR/RR-TB treatment, including significant functional impairments on pulmonary function tests [28, 29], residual chest x-ray abnormalities, and impaired quality of life measures [30]. This review has focused on HIV comorbidity due to the frequency of MDR/RR-TB and HIV co-infection in some parts of the world and to the complexities of treating these co-infections.

Only six published studies reported HIV measures that were repeated after MDR/RR-TB treatment initiation, and all six of these were generally ranked as low-quality evidence on the Johanna Briggs scale [19]. In our search of published literature, most articles reported HIV measures at baseline only and assessed these as co-variates that affect MDR/RR-TB treatment outcomes. Those articles that did report HIV disease-specific outcomes after MDR/RR-TB treatment initiation reported them as secondary outcomes in studies for which the primary outcome was related to MDR/RR-TB. All six studies that reported HIV indicators after MDR/RR-TB initiation were conducted prior to the introduction of all-oral MDR/RR-TB regimens and before WHO recommendations for routine viral load measurement. Therefore, conclusions drawn from the available evidence must be considered in light of the newer MDR/RR-TB and HIV treatment guidelines.

Among the studies that reported repeated measures of HIV disease-specific indicators, CD4 and HIV viral load were the two indicators used. However, when reported, most studies were not explicit in stating the proportion of HIV-positive participants in whom a result was available. Drawing meaningful conclusions from this information is therefore difficult.

Three of the four included cohort studies reported repeated HIV viral load measures. Because WHO has recommended routine viral load monitoring for all people living with HIV only since 2013 and all of the manuscripts returned in the initial search reported outcomes of MDR/RR-TB cohorts who finished treatment in 2013 or before [31], it is unsurprising that only one of the included articles specified that routine viral load monitoring was occurring in that setting during the time period of the study [15]. However, viral load monitoring is the gold standard for determining the efficacy of ART treatment and following the progression of HIV disease [31, 32]. Without routine viral load monitoring, drawing conclusions from these older cohorts about the overall effects of MDR/RR-TB on HIV disease is also difficult.

Given the renewed focus on MDR/RR-TB disease following the release of new guidelines that have revolutionized treatment, the lack of attention to HIV disease indicators, particularly viral load, in recent MDR/RR-TB cohorts is concerning. Each cohort of MDR/RR-TB patients considered by the WHO Guideline Development Group included HIV co-infected individuals, though in two of these cohorts fewer than five percent of participants were HIV positive. None of these recent studies reported any indicator of HIV disease after MDR/RR-TB initiation, so they do not contribute to our understanding of the effects of MDR/RR-TB treatment on HIV disease. However, as routine viral load monitoring is now widely available globally, reporting viral load measures taken after MDR/RR-TB treatment initiation should be possible in most cases [31, 32]. This additional evidence could increase the understanding of how MDR/RR-TB treatment affects HIV disease.

Few of the included studies discussed ART regimen choices. Those that did offer limited insight, simply reporting that HIV co-infected patients were treated according to national HIV guidelines with the regimen available as first-line ART for the country [13–15, 18]. Just as treatment guidelines for MDR/RR-TB have recently been updated, HIV treatment guidelines and recommendations on selection of individual antiretroviral agents change over time. Although the 2016 WHO treatment guidelines for HIV recommend integrase strand transfer inhibitors such as dolutegravir as an option for first-line HIV therapy in adults [32], use of these newer antiretroviral agents was not widespread in any of the countries where these MDR/RR-TB studies were conducted. South Africa is actively transitioning to a dolutegravir-based first-line ART regimen, becoming the first low- or middle-income country in the world to roll out this change at a national level [33]. As ART regimens and MDR/RR-TB regimens are revised over time, greater attention to the identification of ART regimens as well as the regimen’s potential interactions with MDR/RR-TB treatment is imperative. The reporting of HIV viral suppression rates at MDR/RR-TB treatment initiation and completion is essential is essential to evaluate the overall effectiveness of both HIV and MDR/RR-TB treatment regimens and treatment programs.

There are limitations to this literature review. In each of the included articles, HIV disease indicators were reported as secondary results of studies primarily focused on MDR/RR-TB treatment. As participants in each included study were recruited to answer specific and different research questions than those questions posed in this review, it is possible that selection criteria imposed by each study could have contributed to a study sample whose HIV disease indicators would differ from those of the general MDR/RR-TB and HIV co-infected population. Additionally, the review included only articles published in English. Because of this, we may have missed important articles detailing results from other countries published in other languages, as MDR/RR-TB is a global epidemic and is endemic in non-anglophone countries. Finally, as with any review of literature, it is possible that some studies were missed. Every effort was taken by the authors to conduct a thorough and systematic search of published literature, including consultation with a medical librarian, and all returned articles underwent independent review by two authors in order to minimize this risk.

## Conclusions

MDR/RR-TB is a deadly, debilitating disease, but newer treatment regimens are improving outcomes. Published literature has previously focused on reporting MDR/RR-TB outcomes and assessing interventions to improve these, but attention must shift beyond MDR/RR-TB alone and begin to include important comorbidities such as HIV. The sparse evidence available is quickly becoming obsolete, as all studies that report HIV disease indicators after MDR/RR-TB initiation were conducted before newer, all-oral MDR/RR-TB treatment regimens were implemented. Viral load and CD4 count are routinely monitored in most HIV treatment programs worldwide, including those treating MDR/RR-TB co-infected patients. Therefore, these important HIV indicators should be reported along with MDR/RR-TB outcomes in studies that include MDR/RR-TB and HIV co-infected patients. Unless more evidence on the effect of MDR/RR-TB treatment on HIV disease indicators is made available, clinicians will struggle to manage HIV co-infection. A common core of HIV-specific clinical outcomes along with a standardized frequency of measurement is needed for future studies to improve the opportunity for comparison across regimens and program settings.

## Supporting information

S1 Checklist PRISMA 2009 checklist. (DOC)

S1 Table Search strategy. PubMed, EMBASE, and CINAHL were searched using the terms and Boolean operators detailed below.
(ZIP)
