# Supplementary figures and images for: The effects of MDR/RR-TB treatment on HIV disease: A systematic review of literature
Source: PLoS One. 2021 Mar 5;16(3):e0248174. doi: 10.1371/journal.pone.0248174 (PMC7935310; doi:10.1371/journal.pone.0248174)

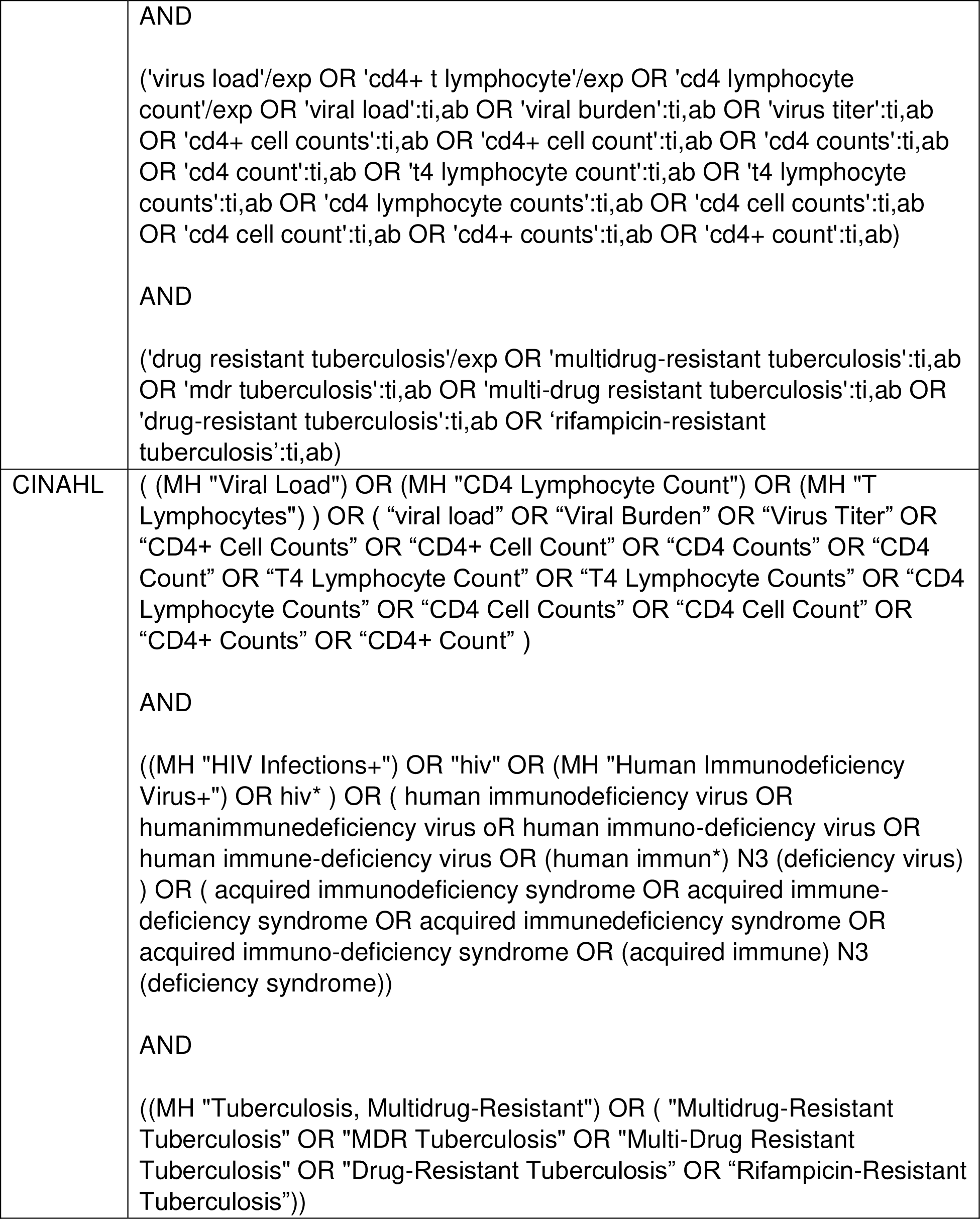

Supplement: S1 Table — PubMed, EMBASE, and CINAHL were searched using the terms and Boolean operators detailed below. (ZIP) [file pone.0248174.s002.zip › S1 Table (3).tif]

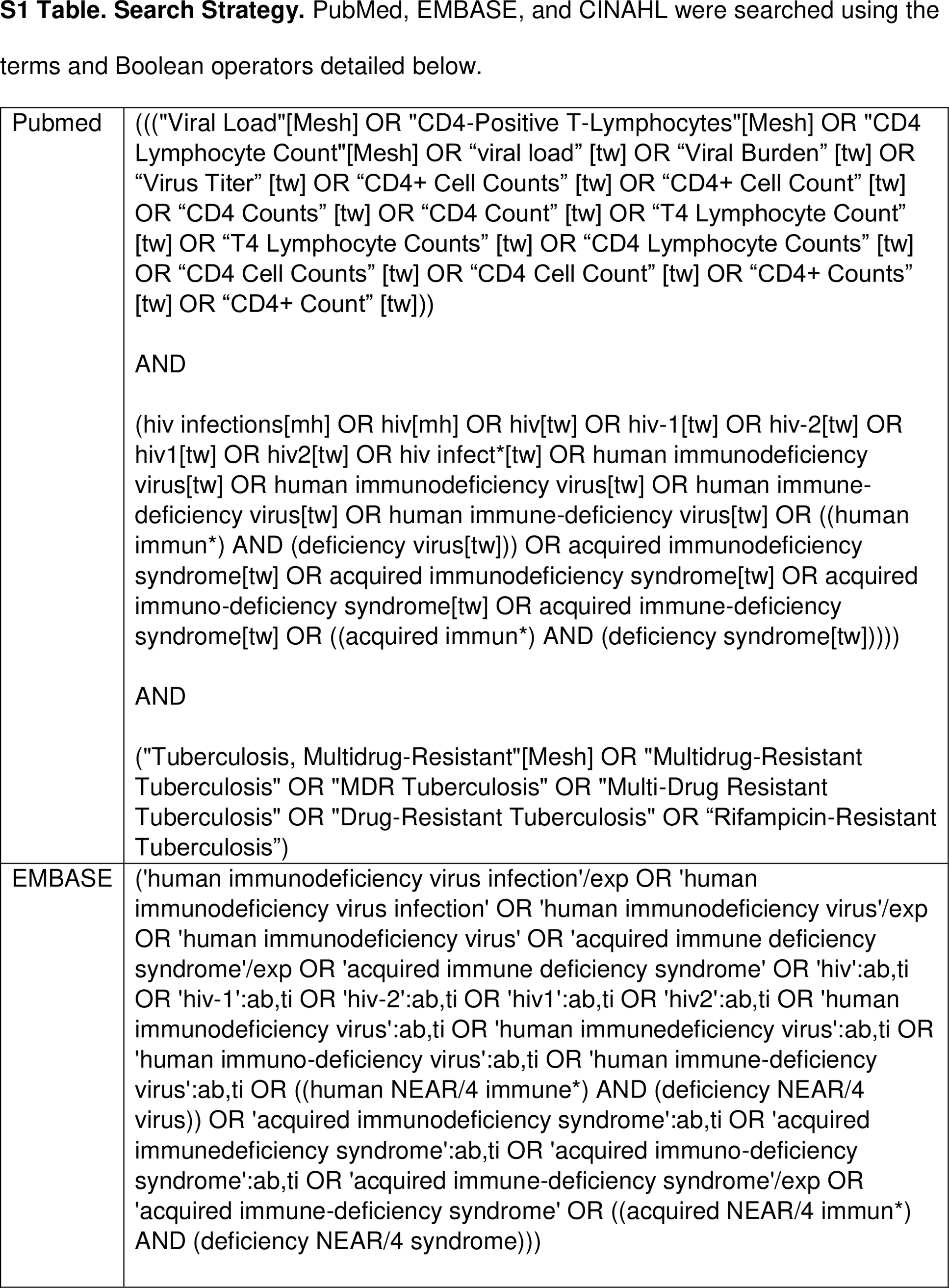

Supplement: S1 Table — PubMed, EMBASE, and CINAHL were searched using the terms and Boolean operators detailed below. (ZIP) [file pone.0248174.s002.zip › S1 Table (4).tif]
